# Hypertension among older adults in low- and middle-income countries: prevalence, awareness and control

**DOI:** 10.1093/ije/dyt215

**Published:** 2014-02-06

**Authors:** Peter Lloyd-Sherlock, John Beard, Nadia Minicuci, Shah Ebrahim, Somnath Chatterji

**Affiliations:** ^1^School of International Development, University of East Anglia, Norwich, UK, ^2^Department of Ageing and Life Course, World Health Organization, Geneva, Switzerland ^3^National Research Council, Institute of Neuroscience, Padova, Italy, ^4^London School of Hygiene and Tropical Medicine, London, UK and ^5^Department of Health Statistics and Informatics, World Health Organization, Geneva, Switzerland

**Keywords:** Hypertension, older people, risk factors, developing countries

## Abstract

**Background** This study uses data from the World Health Organization’s Study on Global Ageing and Adult Health (SAGE) to examine patterns of hypertension prevalence, awareness, treatment and control for people aged 50 years and over in China, Ghana, India, Mexico, the Russian Federation and South Africa.

**Methods** The SAGE sample comprises of 35 125 people aged 50 years and older, selected randomly. Hypertension was defined as ≥140 mmHg (systolic blood pressure) or ≥90 mmHg (diastolic blood pressure) or by currently taking antihypertensives. Control of hypertension was defined as blood pressure below 140/90 mmHg on treatment. A person was defined as aware if he/she was hypertensive and self-reported the condition.

**Results** Prevalence rates in all countries are broadly comparable to those of developed countries (52.9%; range 32.3% in India to 77.9% in South Africa). Hypertension was associated with overweight/obesity and was more common in women, those in the lowest wealth quintile and in heavy alcohol consumers. Awareness was found to be low for all countries, albeit with substantial national variations (48.3%; range 23.3% in Ghana to 72.1% in the Russian Federation). This was also the case for control (10.2%; range 4.1% in Ghana to 14.1% India) and treatment efficacy (26.3%; range 17.4% in the Russian Federation to 55.2% in India). Awareness was associated with increasing age, being female and being overweight or obese. Effective control of hypertension was more likely in older people, women and in the richest quintile. Obesity was associated with poorer control.

**Conclusions** The high rates of hypertension in low- and middle-income countries are striking. Levels of treatment and control are inadequate despite half those sampled being aware of their condition. Since cardiovascular disease is by far the largest cause of years of life lost in these settings, these findings emphasize the need for new approaches towards control of this major risk factor.

## Introduction

Populations around the world are rapidly ageing, and low- and middle-income countries (LMICS) are experiencing some of the most dramatic increases.^1^ This demographic transition is closely linked to an epidemiological shift from communicable to non-communicable disease (NCD).^2^ Hypertension, a key NCD risk factor, appears to be increasing in prevalence, possibly associated with development, urbanization and lifestyle changes.^2^^,^^3^ However, there are large variations in reported prevalence, both across and within countries.^4^^,^^5^ This is particularly apparent in LMICs, although these discrepancies may be partly due to variations in survey design and measurement. Systematically verifying the extent of these national and sub-national variations using directly measured blood pressure, and identifying potentially modifiable causes, may facilitate the development of interventions to slow the rise in NCD occurrence. Systematic analysis is also needed to assess the degree to which hypertension is detected, treated and controlled, and assess social gradients for all these aspects of hypertension.

Hypertension prevalence increases with age, and is a readily treatable risk factor for the most common causes of morbidity and mortality in older age: stroke, ischaemic heart disease, renal insufficiency and dementia.^6–8^ It has been suggested that the burden of stroke and ischaemic heart disease may be several times higher in LMICs than in their high-income counterparts.^2^ Yet, whereas LMICs are experiencing the most rapid population ageing, our understanding of the prevalence and management of hypertension in these settings remains limited. Although there is some evidence of high prevalence in LMICs^9–11^ these studies are small, do not always include older participants, and little is concluded about the awareness of hypertension, the extent of effective or ineffective treatment, or the factors that might influence awareness or treatment in these settings. To fill this crucial gap in our understanding, this study examines the prevalence and possible determinants of hypertension and effective treatment in representative samples of over 35 000 older people in low- and middle-income settings.

## Methods

We use new publicly available data from the World Health Organization (WHO) Study on Global Aging and Adult Health (SAGE). This comprises nationally representative household surveys in China, Ghana, India, Mexico, South Africa and the Russian Federation. Respondents were selected using a multi-stage, stratified, random cluster sampling design with every individual having a known non-zero probability of being selected. The primary sampling units were stratified by region and location (urban/rural) and, within each stratum, enumeration areas were selected. Details are available on the SAGE website (www.who.int/healthinfo/systems/sage). The six countries comprise a total study population of 35 125 people aged 50 years and over. The period of data collection varied by country, ranging between 2007 and 2010. Overall individual response rates among those eligible within households and agreeing to participate in the survey varied from 52.4% in Mexico to 92.3% in China (Table 1). Supplementary Table A1 (available as Supplementary data at *IJE* online) provides more detailed information about response rates to different questionnaire items. Ethical clearance was obtained from local research review boards at each participating SAGE site and the WHO Ethical Review Committee. Informed consent was obtained from each respondent prior to interview. Further information about the SAGE survey design and methods is available from a published data resource profile.^12^

**Table 1:** Distribution (%) of characteristics, overall and by country

		**Overall**	**China**	**Ghana**	**India**	**Mexico**	**Russian Federation**	**South Africa**
		(*n* = 35 125)	(*n* = 13 348)	(*n* = 4716)	(*n* = 7238)	(*n* = 2281)	(*n* = 3763)	(*n* = 3820)
**Age (years)**	50–54	23.1	21.7	21.0	23.5	26.3	23.1	28.3
	55–59	23.6	23.3	19.4	25.3	21.7	22.1	21.5
	60–64	15.7	17.1	14.8	16.5	14.3	11.5	16.9
	65–69	13.8	14.6	12.5	14.0	11.2	13.1	13.7
	70–74	10.5	11.1	14.3	10.6	7.8	10.4	8.3
	75+	13.2	12.1	18.0	10.0	18.6	19.8	11.2
**Sex**	Male	48.0	49.8	49.7	51.1	46.8	38.9	44.0
	Female	52.0	50.2	50.3	48.9	53.2	61.1	56.0
**Education**	None	28.9	23.8	54.0	51.2	17.2	0.7	25.2
	Primary	30.2	39.5	21.3	24.8	62.4	6.8	46.4
	Secondary	15.5	19.7	4.0	10.2	9.9	20.2	14.2
	Higher	25.4	17.0	20.7	13.7	10.5	72.3	14.2
**BMI**	Normal	45.9	60.5	55.3	48.3	21.4	23.8	24.7
	Underweight	15.6	4.3	15.3	39.0	0.6	1.1	3.3
	Overweight	26.1	29.5	19.7	10.6	49.4	40.8	26.9
	Obese	12.4	5.7	9.7	2.1	28.6	34.3	45.1
**Smoking**	Never	64.6	64.2	77.3	61.6	60.6	69.6	69.0
	Less than daily	3.0	2.5	2.7	3.9	6.9	1.2	3.4
	Daily	24.1	26.7	5.4	28.1	13.3	20.1	17.8
	Ever (not current)	8.2	6.6	14.6	6.3	19.1	9.0	9.7
**Alcohol consumption**	Life time abstainers	76.8	74.2	57.8	92.5	64.3	44.7	84.5
	Non-heavy drinker	18.4	18.2	39.5	6.9	29.3	47.6	11.5
	Infrequent heavy drinker	1.9	1.2	1.2	0.4	6.2	6.3	3.0
	Frequent heavy drinker	2.8	6.3	1.5	0.2	0.1	1.4	1.0
**Physical activity**	High	48.7	44.1	61.7	52.2	39.6	57.7	28.0
	Moderate	22.8	27.5	12.6	22.9	22.8	15.7	12.6
	Low	28.5	28.3	25.7	24.9	37.6	26.6	59.4
**Location**	Urban	48.1	47.6	40.6	29.4	78.8	72.7	64.9
	Rural	51.9	52.4	59.4	70.6	21.2	27.3	35.1
**Wealth quintile**	Poorest	17.0	16.3	18.4	17.9	15.3	16.2	20.7
	Q2	19.4	18.2	19.4	19.3	24.7	19.6	19.9
	Q3	19.3	20.5	20.7	18.8	16.8	19.1	18.2
	Q4	20.8	23.3	20.0	19.5	16.6	20.5	19.8
	Richest	23.5	21.7	21.5	24.4	26.6	24.6	21.4
**Health insurance**	Insured	56.0	89.6	38.0	3.9	66.6	99.6	20.5
	Uninsured	44.0	10.4	62.0	96.1	33.4	0.4	79.5
**Response rate (%)**			92.3	78.4	87.3	52.4	82.3	77.7

Blood pressure (BP) was measured using a Boso Medistar Wrist Blood Pressure Monitor Model S. Validation studies of similar wrist blood pressure monitoring devices indicate that they are capable of providing accurate measurements^13^^,^^14^ but that the position of the arm in relation to the heart is critical (http://www.bhsoc.org/bp-monitors/bp-monitors/).

Respondents were asked to remain seated and relaxed, and the importance of positioning their arm level with their heart was emphasized. BP was measured three times, with 1 min between each measurement. Participants were asked to self-report: ‘Have you ever been diagnosed with high BP (hypertension)?’ Those answering ‘yes’ were further asked: ‘Have you been taking any medications or other treatment for it during a) the last 2 weeks? b) the last 12 months?’ Height was measured using a stadiometer and weight was measured using an electronic weighing scale that was periodically calibrated. All interviewers were provided with a week of training, including demonstrations and audiovisual aids. Before launching the main phase of the study, a pretest was carried out on 1446 respondents, with 5% of respondents being retested within 7 days with an identical instrument. The SAGE main study also included a retest component on 5% of respondents. These studies show a moderate reliability in these measures with trained lay interviewers (kappa for obesity 0.8; for hypertension 0.5).

Hypertension was considered to be present if the mean of the last two measurements was ≥140 mmHg (systolic blood pressure) or ≥90 mmHg (diastolic blood pressure) or if respondents were currently taking antihypertensives. Hypertensive participants were classified as controlled (i.e. on antihypertensives and blood pressure <140 or <90 mmHg). Those who were aware of their hypertension were classified into controlled and uncontrolled. Finally, among all those who were currently on treatment for hypertension, respondents were classified into those who were effectively treated (normotensive on measurement) and those who were not effectively treated (hypertensive on measurement).

All data analysis was weighted, using individual weights that were post-stratified based on the UN Population Standards data.^15^ All analyses were age standardized. Analysis was carried out using SAS 9.3 (Statistical Analysis Software, SAS Institute). Multivariable logistic regression models were used to examine the association of a range of characteristics with the following outcomes: hypertension, awareness of hypertension, use of antihypertensives and control. Models were applied to each country sample individually, and to the total pooled sample. All characteristics were retained in the final ‘forced’ multivariable model, with complete data on all variables. The adjusted model included: age, sex, education, body mass index (BMI, kg/m), location (rural/urban), wealth quintiles at the household level, and household head’s membership of a health insurance scheme. Details of classification of variables are presented in Appendix 1 (available as Supplementary data at *IJE* online). For those receiving treatment, we applied similar models (adding inadequate physical activity and heavy alcohol consumption) to determine the factors that were associated with effective treatment (i.e. treated but with normal measured blood pressure) compared with ineffective treatment (detailed definitions of these variables are provided in Appendix 1, available as Supplementary data at *IJE* online).

## Results

Table 1 shows the distribution of variables potentially associated with hypertension by country and for the SAGE sample as a whole. Table 2 shows there were high rates of hypertension for all the SAGE countries, albeit with large national variations. The prevalence of hypertension ranged from 78% in South Africa to 32% in India, with consistently higher levels for women.

**Table 2:** Prevalence and odds ratios (95% CI) for hypertension by various characteristics across all sites; odds ratios are adjusted for all of the other variables in the table

		Overall	China	Ghana	India	Mexico	Russian Federation	South Africa
		(*n* = 27 376)	(*n* = 11 964)	(*n* = 3923)	(*n* = 4474)	(*n* = 2010)	(*n* = 3191)	(*n* = 2583)
**Prevalence of Hypertension (%)**								
	Total	52.9 (52.3–53.4)	59.5 (58.4–60.6)	57.1 (55.1–59.1)	32.3 (30.3–34.3)	58.2 (55.4–61.0)	71.7 (69.9–73.5)	77.9 (76.4–79.4)
	Male	49.5 (48.7–50.3)	58.8 (57.6–60.0)	54.6 (52.5–56.7)	30.3 (28.7–31.9)	55.2 (52.1–58.3)	65.9 (63.4–68.4)	74.7 (72.6–76.8)
	Female	55.9 (55.1–56.6)	60.1 (58.9–61.3)	59.9 (57.8–62.0)	35.0 (33.3–36.7)	60.9 (58.0–63.8)	74.5 (72.6–76.4)	80.3 (78.6–82.0)
**Odds Ratios**								
**Age (years)**	50–54	1	1	1	1	1	1	1
	55–59	1.44 (1.34–1.55)	1.30 (1.16–1.45)	1.12 (0.91–1.38)	1.60 (1.32–1.94)	1.69 (1.25–2.27)	1.57 (1.24–1.99)	1.31 (1.00–1.73)
	60–64	1.79 (1.65–1.95)	1.76 (1.56–1.99)	1.24 (0.99–1.56)	1.51 (1.22–1.88)	2.52 (1.79–3.54)	2.45 (1.80–3.33)	1.53 (1.13–2.07)
	65–69	2.26 (2.07–2.47)	2.29 (2.00–2.61)	1.53 (1.21–1.95)	2.03 (1.62–2.54)	4.52 (3.02–6.76)	2.13 (1.60–2.83)	1.50 (1.07–2.09)
	70–74	2.80 (2.53–3.09)	2.83 (2.43–3.29)	1.32 (1.04–1.67)	2.20 (1.71–2.82)	4.73 (2.97–7.52)	4.04 (2.84–5.74)	1.53 (1.02–2.29)
	75+	3.67 (3.32–4.05)	3.47 (2.96–4.07)	1.28 (1.02–1.61)	2.34 (1.79–3.06)	5.42 (3.72–7.09)	6.81 (4.83–9.60)	1.83 (1.25–2.68)
**Sex**	Male	1	1	1	1	1	1	1
	Female	1.16 (1.08–1.24)	0.94 (0.85–1.05)	1.16 (1.00–1.35)	1.38 (1.15–1.65)	1.17 (0.92–1.48)	1.17 (0.94–1.46)	1.29 (1.05–1.59)
**Education**	Primary	1	1	1	1	1	1	1
	None	0.93 (0.87–1.00)	0.96 (0.86–1.07)	0.82 (0.69–0.98)	1.03 (0.87–1.23)	1.68 (1.21–2.33)	0.17 (0.05–0.55)	1.11 (0.85–1.43)
	Secondary	1.01 (0.93–1.09)	1.03 (0.93–1.15)	0.76 (0.53–1.10)	1.09 (0.85–1.40)	0.53 (0.37–0.76)	0.56 (0.34–0.91)	0.77 (0.57–1.04)
	Higher	0.94 (0.87–1.01)	0.85 (0.75–0.96)	0.96 (0.78–1.18)	1.06 (0.84–1.34)	0.70 (0.49–1.00)	0.52 (0.33–0.84)	0.67 (0.48–0.93)
**BMI**	Normal	1	1	1	1	1	1	1
	Underweight	0.44 (0.40–0.48)	0.47 (0.39–0.57)	0.65 (0.54–0.79)	0.54 (0.47–0.64)	0.43 (0.11–1.67)	0.27 (0.13–0.59)	0.96 (0.58–1.59)
	Overweight	1.91 (1.79–2.03)	2.02 (1.85–2.21)	1.77 (1.48–2.12)	1.55 (1.26–1.92)	2.83 (2.14–3.74)	1.77 (1.44–2.16)	1.57 (1.20–2.06)
	Obese	4.09 (3.73–4.49)	2.74 (2.28–3.30)	2.30 (1.76–3.00)	1.86 (1.25–2.77)	3.38 (2.47–4.62)	7.05 (5.50–9.03)	1.85 (1.43–2.39)
**Smoking**	Never	1	1	1	1	1	1	1
	Less than daily/ever (not current)	1.11 (1.01–1.22)	1.14 (0.98–1.34)	1.24 (1.02–1.49)	1.20 (0.95–1.51)	1.32 (1.01–1.74)	0.75 (0.55–1.02)	1.05 (0.78–1.42)
	Daily	0.96 (0.89–1.03)	0.82 (0.72–0.92)	0.83 (0.61–1.12)	0.97 (0.80–1.17)	0.82 (0.60–1.13)	1.25 (0.97–1.63)	1.07 (0.81–1.42)
**Alcohol consumption**	Life-time abstainers	1	1	1	1	1	1	1
	Non-heavy drinker	1.05 (0.98–1.14)	1.02 (0.91–1.15)	0.80 (0.69–0.94)	1.39 (1.03–1.87)	0.34 (0.26–0.45)	1.34 (1.09–1.65)	0.66 (0.47–0.91)
	Infrequent heavy drinkers	1.07 (0.87–1.31)	1.10 (0.77–1.59)	0.80 (0.40–1.59)	1.53 (0.50–4.69)	1.66 (1.00–2.77)	1.02 (0.65–1.59)	0.41 (0.25–0.67)
	Frequent heavy drinkers	1.88 (1.58–2.23)	1.72 (1.43–2.07)	1.24 (0.66–2.33)	7.46 (1.49–37.3)	Not estimable	0.77 (0.34–1.76)	0.45 (0.17–1.15)
**Physical activity**	High	1	1	1	1	1	1	1
	Moderate	1.06 (0.99–1.13)	1.05 (0.95–1.16)	1.02 (0.83–1.25)	0.99 (0.83–1.17)	1.66 (1.26–2.20)	1.07 (0.84–1.37)	0.91 (0.66–1.26)
	Low	1.13 (1.06–1.21)	1.06 (0.96–1.17)	1.09 (0.92–1.30)	1.21 (1.03–1.43)	1.15 (0.89–1.48)	1.07 (0.85–1.34)	0.77 (0.61–0.97)
**Location**	Urban	1	1	1	1	1	1	1
	Rural	1.11 (1.05–1.18)	1.35 (1.22–1.49)	0.73 (0.63–0.85)	1.13 (0.97–1.32)	0.97 (0.74–1.27)	0.92 (0.75–1.13)	1.04 (0.83–1.31)
**Wealth quintile**	Poorest	1	1	1	1	1	1	1
	Q2	0.98 (0.90–1.07)	0.88 (0.77–1.00)	1.11 (0.90–1.38)	0.97 (0.77–1.23)	2.93 (2.04–4.21)	1.16 (0.87–1.56)	0.87 (0.64–1.17)
	Q3	0.92 (0.84–1.01)	0.90 (0.78–1.02)	1.77 (0.95–1.45)	1.09 (0.87–1.37)	0.92 (0.62–1.36)	1.27 (0.94–1.70)	1.03 (0.75–1.42)
	Q4	0.98 (0.90–1.07)	1.00 (0.87–1.14)	1.77 (0.94–1.47)	1.12 (0.89–1.41)	1.16 (0.78–1.71)	1.51 (1.13–2.02)	1.26 (0.90–1.78)
	Richest	0.86 (0.79–0.94)	0.93 (0.81–1.08)	1.16 (0.91–1.48)	1.44 (1.15–1.82)	1.43 (0.98–2.09)	0.77 (0.58–1.01)	1.26 (0.88–1.80)
**Health insurance**	Insured	1	1	1	1	1	1	1
	Uninsured	0.55 (0.52–0.59)	0.85 (0.75–0.97)	1.08 (0.94–1.25)	0.71 (0.52–0.98)	0.86 (0.69–1.08)	2.46 (0.62–9.67)	0.92 (0.71–1.20)

Adjusted individual-level multivariable analysis showed positive associations with increasing age and body mass index (BMI) consistently across countries. Women had higher odds of hypertension in all countries but China. Increasing BMI was strongly associated with hypertension. This was consistent across countries, and the large variations in the prevalence of overweight/obesity across the SAGE countries (16.6% in India, 83.5% in the Russian Federation) were a key determinant for the national variations in prevalence reported in Table 2. The inverse association with higher education was found for all countries except India, where education had no effect, and Ghana, where only people with no education were less likely to have hypertension. The effects of smoking and physical exercise were inconsistent across countries and point estimates were small, with wide confidence intervals. The effect of alcohol consumption was very inconsistent, which partly reflects the very low numbers reporting heavy drinking.

There were large national variations in the proportion of hypertensive participants who were aware of their condition (Table 3) and who were adequately controlled (Table 4). The Russian Federation was the only country with a high percentage of people aware of their status (72%); in none of the other five countries were more than 45% of hypertensive people aware. Factors consistently associated with awareness across the six countries included increasing age, being female and being overweight or obese. Having health insurance was positively associated with awareness in three countries, but the strength of this effect varied from very strong in the Russian Federation to more marginal in Ghana. With the exception of Mexico and the Russian Federation, urban residence was associated with awareness. Higher wealth quintiles were associated with awareness in every country other than Mexico. The effect of higher education status was less consistent, with positive associations in four countries.

**Table 3:** Prevalence of awareness and adjusted model for being aware of the presence of hypertension by country (odds ratios with 95% confidence intervals)

		Overall	China	Ghana	India	Mexico	Russian Federation	South Africa
		(*n* = 17396)	(*n* = 7167)	(*n* = 2314)	(*n* = 1962)	(*n* = 1159)	(*n* = 2260)	(*n* = 2147)
**Prevalence of Awareness (%)**								
**Total**		48.3 (47.5–49.0)	42.7 (41.6–43.8)	23.3 (21.6–25.0)	37.8 (35.7–39.9)	44.6 (41.8–47.4)	72.1 (70.3–73.9)	38.0 (36.2–39.8)
**Male**		41.5 (40.4–42.5)	38.0 (36.4–39.6)	19.2 (17.0–21.4)	35.6 (32.6–38.6)	39.3 (35.2–43.4)	61.4 (58.2–64.6)	32.8 (30.1–35.5)
**Female**		53.9 (52.9–54.8)	47.2 (45.6–48.8)	27.5 (25.0–30.0)	39.9 (36.9–42.9)	48.7 (44.9–52.5)	78.2 (76.2–80.2)	41.8 (39.4–44.2)
**Odds Ratios**								
**Age (years)**	50–54	1	1	1	1	1	1	1
	55–59	1.30 (1.17–1.44)	1.33 (1.13–1.57)	1.29 (0.91–1.83)	1.12 (0.83–1.52)	6.47 (3.85–10.8)	0.99 (0.73–1.35)	1.71 (1.30–2.25)
	60–64	1.42 (1.27–1.59)	1.65 (1.39–1.97)	1.46 (1.00–2.13)	0.93 (0.65–1.32)	7.69 (4.50–13.1)	0.85 (0.60–1.20)	1.65 (1.23–2.20)
	65–69	2.03 (1.81–2.27)	2.30 (1.93–2.75)	2.00 (1.38–2.90)	1.43 (1.02–1.99)	6.83 (3.99–11.6)	1.32 (0.93–1.89)	2.93 (2.17–3.95)
	70+	2.56 (2.31–2.83)	2.36 (1.99–2.79)	2.49 (1.79–3.47)	2.13 (1.56–2.92)	8.69 (5.32–14.1)	1.62 (1.19–2.21)	2.60 (1.97–3.42)
**Sex**	Male	1	1	1	1	1	1	1
	Female	1.72 (1.61–1.84)	1.39 (1.25–1.54)	2.00 (1.59–2.50)	1.79 (1.40–2.27)	1.95 (1.47–2.57)	2.37 (1.94–2.89)	1.58 (1.30–1.91)
**BMI**	Normal	1	1	1	1	1	1	1
	Underweight	0.61 (0.53–0.70)	0.70 (0.51–0.96)	0.78 (0.53–1.15)	0.50 (0.39–0.65)	1.39 (0.17–11.4)	0.24 (0.07–0.87)	1.84 (1.07–3.16)
	Overweight/ Obese	1.83 (1.70–1.96)	1.70 (1.54–1.89)	1.57 (1.25–1.98)	1.98 (1.53–2.56)	2.26 (1.59–3.22)	1.83 (1.44–2.32)	1.50 (1.18–1.90)
**Health insurance**	Insured	1	1	1	1	1	1	1
	Uninsured	0.81 (0.75–0.87)	0.93 (0.78–1.11)	0.67 (0.54–0.83)	0.70 (0.45–1.07)	0.41 (0.30–0.56)	0.19 (0.05–0.70)	0.83 (0.66–1.06)
**Location**	Urban	1	1	1	1	1	1	1
	Rural	0.74 (0.69–0.79)	0.44 (0.39–0.49)	0.59 (0.47–0.74)	0.72 (0.58–0.90)	1.02 (0.73–1.43)	1.19 (0.94–1.50)	0.77 (0.62–0.95)
**Education**	Primary	1	1	1	1	1	1	1
	None	0.90 (0.82–0.99)	1.01 (0.88–1.16)	0.66 (0.50–0.88)	0.80 (0.61–1.04)	1.03 (0.71–1.50)	2.78 (0.63–12.3)	0.93 (0.74–1.18)
	Secondary/ higher	2.04 (1.88–2.20)	1.31 (1.16–1.48)	1.51 (1.12–2.02)	2.10 (1.57–2.81)	1.12 (0.76–1.65)	1.56 (1.07–2.26)	0.82 (0.65–1.03)
**Wealth quintile**	Poorest	1	1	1	1	1	1	1
	Q2	1.13 (1.01–1.26)	1.15 (0.97–1.37)	1.01 (0.64–1.58)	1.94 (1.31–2.88)	0.39 (0.25–0.60)	1.70 (1.24–2.32)	1.05 (0.78–1.42)
	Q3	1.32 (1.18–1.47)	1.20 (1.01–1.43)	1.39 (0.91–2.13)	1.61 (1.08–2.39)	0.90 (0.55–1.46)	1.98 (1.44–2.72)	1.35 (1.00–1.82)
	Q4	1.39 (1.25–1.55)	1.30 (1.09–1.54)	1.98 (1.31–3.00)	1.99 (1.35–2.92)	0.88 (0.54–1.44)	1.57 (1.16–2.13)	1.28 (0.94–1.75)
	Richest	1.76 (1.58–1.97)	1.32 (1.10–1.59)	2.35 (1.53–3.60)	2.08 (1.41–30.6)	0.97 (0.60–1.56)	3.30 (2.35–4.62)	1.60 (1.16–2.21)

**Table 4:** Prevalence of control and adjusted model for effective control of hypertension in hypertensive participants (odds ratios with 95% confidence intervals)

		Overall	China	Ghana	India	Mexico	Russian Federation	South Africa
		(n = 17 366)	(n = 7152)	(n = 2303)	(n = 1962)	(n = 1159)	(n = 2256)	(n = 2129)
**Prevalence of Control (%)**								
**Total**		10.2 (9.7–10.6)	8.3 (7.7–8.9)	4.1 (3.3–4.9)	14.1 (12.6–15.6)	11.8 (10.0–13.6)	10.5 (9.3–11.7)	7.8 (6.8–8.8)
**Male**		9.1 (8.5–9.7)	8.2 (7.3–9.1)	4.0 (2.9–5.1)	13.1 (11.0–15.2)	6.5 (4.4–8.6)	8.8 (7.0–10.6)	5.8 (4.5–7.1)
**Female**		11.1 (10.5–11.7)	8.3 (7.4–9.2)	4.3 (3.2–5.4)	15.1 (12.9–17.3)	15.9 (13.2–18.6)	11.5 (9.9–13.1)	9.2 (7.8–10.6)
**Odds Ratios**								
**Age (years)**	50–54	1	1	1	1	1	1	1
	55–59	1.45 (1.22–1.73)	1.39 (1.01–1.93)	1.93 (0.86–4.32)	1.30 (0.85–2.00)	11.7 (4.42–31.2)	0.99 (0.60–1.61)	1.39 (0.80–2.38)
	60–64	1.49 (1.23–1.80)	1.40 (1.01–1.96)	0.95 (0.34–2.66)	1.32 (0.80–2.15)	4.56 (1.62–12.8)	1.71 (1.03–2.84)	0.69 (0.36–1.33)
	65–69	1.78 (1.48–2.14)	1.72 (1.25–2.39)	2.69 (1.17–6.15)	1.49 (0.92–2.42)	4.91 (1.76–13.7)	1.60 (0.96–2.66)	3.11 (1.88–5.16)
	70+	1.82 (1.53–2.16)	1.53 (1.12–2.09)	2.60 (1.20–5.62)	2.47 (1.58–3.86)	5.74 (2.21–14.8)	1.18 (0.72–1.93)	1.56 (0.93–2.61)
**Sex**	Male	1	1	1	1	1	1	1
	Female	1.22 (1.09–1.36)	0.86 (0.71–1.04)	1.36 (0.85–2.18)	1.41 (1.01–1.96)	2.66 (1.64–4.31)	1.79 (1.29–2.49)	1.70 (1.16–2.48)
**BMI**	Normal	1	1	1	1	1	1	1
	Underweight	0.83 (0.66–1.03)	0.81 (0.45–1.47)	0.59 (0.23–1.53)	0.72 (0.49–1.03)	3.97 (0.36–42.9)	<0.001 (<0.001- >999)	5.52 (2.38–12.7)
	Overweight	0.95 (0.84–1.07)	0.93 (0.77–1.12)	0.79 (0.47–1.34)	1.37 (0.97–1.93)	1.45 (0.82–2.56)	0.67 (0.47–0.97)	0.99 (0.56–1.74)
	Obese	0.81 (0.69–0.94)	0.90 (0.63–1.28)	0.68 (0.36–1.29)	1.71 (0.94–3.11)	1.14 (0.61–2.12)	0.50 (0.33–0.74)	1.50 (0.90–2.49)
**Health insurance**	Insured	1	1	1	1	1	1	1
	Uninsured	1.44 (1.28–1.62)	1.16 (0.90–1.51)	0.67 (0.43–1.05)	0.49 (0.31–0.78)	0.34 (0.19–0.59)	0.67 (0.07–6.41)	0.80 (0.53–1.22)
**Location**	Urban	1	1	1	1	1	1	1
	Rural	0.51 (0.45–0.58)	0.17 (0.13–0.22)	0.42 (0.23–0.74)	0.73 (0.54–0.97)	1.52 (0.94–2.45)	0.57 (0.40–0.83)	0.74 (0.49–1.12)
**Education**	Primary	1	1	1	1	1	1	1
	None	1.15 (0.98–1.34)	1.01 (0.76–1.33)	0.77 (0.42–1.41)	1.08 (0.75–1.56)	2.01 (1.16–3.48)	0.17 (0.01–5.19)	1.63 (1.05–2.53)
	Secondary/ higher	1.40 (1.24–1.59)	1.39 (1.13–1.71)	1.10 (0.61–2.01)	1.46 (0.99–2.15)	1.22 (0.68–2.20)	0.73 (0.43–1.24)	1.34 (0.88–2.03)
**Wealth quintile**	Poorest	1	1	1	1	1	1	1
	Q2	1.14 (0.93–1.40)	1.22 (0.85–1.74)	0.50 (0.14–1.69)	1.37 (0.71–2.62)	0.48 (0.23–1.01)	1.36 (0.79–2.35)	3.03 (1.54–5.96)
	Q3	1.41 (1.16–1.73)	1.24 (0.88–1.75)	0.93 (0.33–2.64)	1.51 (0.79–2.88)	1.39 (0.68–2.82)	1.36 (0.79–2.36)	2.11 (1.04–4.31)
	Q4	2.02 (1.67–2.44)	1.30 (0.93–1.83)	1.17 (0.44–3.11)	2.88 (1.60–5.20)	1.77 (0.87–3.60)	2.23 (1.32–3.77)	3.64 (1.83–7.27)
	Richest	2.28 (1.89–2.75)	1.00 (0.70–1.42)	2.83 (1.10–7.28)	3.11 (1.71–5.63)	1.29 (0.65–2.58)	2.51 (1.48–4.25)	3.91 (1.94–7.85)
**Alcohol consumption**	Life-time abstainers	1	1	1	1	1	1	1
	Non heavy drinker	0.78 (0.66–0.91)	0.70 (0.53–0.94)	1.57 (0.94–2.63)	0.52 (0.25–1.07)	0.47 (0.19–1.15)	1.30 (0.93–1.83)	0.28 (0.10–0.75)
	Infrequent heavy drinkers	0.88 (0.59–1.31)	0.62 (0.23–1.64)	<0.001 (<0.001- >999)	<0.001 (<0.001- >999)	0.22 (0.05–0.89)	2.34 (1.14–4.80)	0.80 (0.21–3.04)
	Frequent heavy drinkers	0.34 (0.19–0.61)	0.53 (0.28–0.97)	<0.001 (<0.001- >999)	<0.001 (<0.001- >999)	<0.001 (<0.001- >999)	0.10 (<0.001–65.3)	<0.001 (<0.001- >999)
**Physical activity**	High	1	1	1	1	1	1	1
	Moderate	1.17 (1.03–1.32)	1.31 (1.06–1.62)	1.98 (1.00–3.92)	0.91 (0.65–1.28)	1.41 (0.81–2.45)	1.05 (0.71–1.55)	2.29 (1.26–4.16)
	Low	0.91 (0.80–1.04)	1.12 (0.90–1.41)	2.78 (1.65–4.68)	0.74 (0.52–1.05)	1.02 (0.61–1.70)	0.74 (0.50–1.10)	2.18 (1.36–3.49)

Table 4 shows large national variations in the proportion of hypertensive subjects whose condition was effectively controlled, ranging from 4% in Ghana to 14% in India, and the extent to which awareness led to control, ranging from 18% in Ghana to 37% in India. Our adjusted model shows that older age was associated with higher rates of control in five of the six countries, and female sex, urban residence and higher wealth quintiles in four countries. Being overweight or obese had no consistent effect on control across the study countries, except in the Russian Federation. Health insurance was positively associated with control in just two of the six countries. Supplementary Table A2 (available as Supplementary data at *IJE* online) shows large national variations in the effective treatment of blood pressure among people taking antihypertensive medication, ranging from 24% in South Africa to 55% in India. Supplementary Table A2 (available as Supplementary data at *IJE* online) shows that effective treatment of blood pressure was inversely associated with increasing BMI in three of the six countries. For the other factors there was no consistent pattern of association across the SAGE countries.

**Figure 1:**
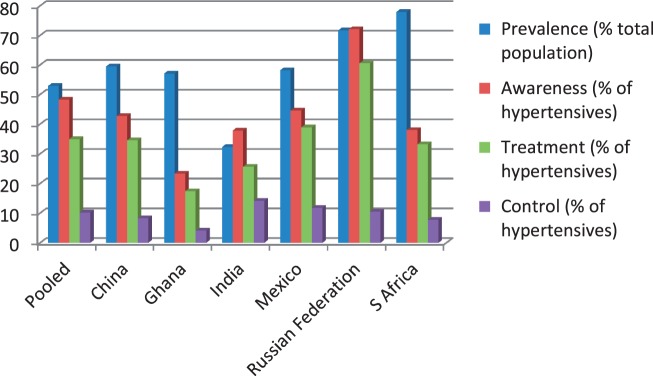
Summaries of the national data for hypertension, prevalence, awareness and treatment, as presented in the main data tables

Figure 1 summarizes the national data for hypertension, prevalence, awareness and treatment, as presented in the main data tables.

## Discussion

This study examines the factors associated with prevalence, awareness and treatment of hypertension for large nationally representative samples of older people in LMICs. This analysis focuses on people over the age of 50 years, since people over this threshold have markedly increased risks of cardiovascular disease (CVD) and will derive greatest benefit from drug treatment in terms of numbers needed to treat. WHO guidelines for prevention of CVD also indicate that people aged 50 and over are the age range of highest risk and therefore of relevance for intervention with a polypill.^16^ Preventive measures such as attempts to reduce salt use in the whole population will clearly have impact on people at younger as well as at older ages. WHO SAGE includes a comparison group of 7344 people aged between 18 and 49 years. A separate analysis of hypertension for the SAGE population as a whole reports similar levels of prevalence, awareness, treatment and control to those reported here.^17^

We found high prevalences of hypertension in all countries, with the highest being 78% for South Africa. Around half those sampled were aware of their condition, but only a very small proportion (4.1% to 14.1%) achieved blood pressure control. The prevalence levels of hypertension observed in this study are broadly consistent with those reported by previous national surveys of older adults conducted in China and Mexico.^18^^,^^19^ National hypertension survey data have not previously been available for India, the Russian Federation or Ghana, either for their adult populations in general or for older people more specifically. A 2003 national survey in South Africa, using similar methodology to SAGE, found a prevalence of hypertension of only 31% for men aged 65 years and over and 37% for women in the same age group.^20^ Reported rates had fallen sharply since the previous 1998 survey (52 and 60%, respectively). The survey authors themselves doubt the credibility of their finding, and report that it is likely that measurements were incorrectly taken by fieldworkers. With the exception of India, the prevalence of hypertension in the SAGE countries is comparable to that of older people in high-income countries.^21–23^ Indeed, South Africa’s prevalence is the highest ever reported by a nationally representative survey of people aged 50 and over for any country. It is substantially higher than recently published estimates for South Africa and 11 other sub-Saharan African countries.^24^

In common with most epidemiological assessments of hypertension prevalence, blood pressure was measured thrice, at intervals of 1 min. In clinical practice blood pressure would be measured on at least three separate occasions spaced over longer periods of time before a diagnosis of hypertension was made and, if this is done, lower prevalences will be found due to regression to the mean.^25^^,^^26^ However, measurements made within a single measurement session do have strong predictive power for cardiovascular disease.^27^

For all six countries, prevalence increased sharply with age, although this effect was more notable in some countries (such as Mexico) than others (such as Ghana). This is in keeping with the findings of the other national studies in China and Mexico.^18^^,^^19^ The observed international variation suggests that, although chronological age is a significant risk factor, increases with age are not inevitable, indicating that age may be acting as a marker of long-term exposure to other risk factors. For five of the six countries, prevalence was higher for women than for men. The consistency of this finding is at odds with other studies from LMICs which show varied gender effects.^28–31^ It is also at odds with a systematic meta-analysis of global blood pressure trends, which showed that men had higher average blood pressure than women in all world regions other than West Africa.^32^

Although only a few studies have examined awareness, treatment and control for older people in LMICs, the findings that are available broadly concur with those we report. For example, the 2000/01 China national survey reported that 46% of people aged 60 years and over were aware of their condition and 9% had it controlled.^18^ Studies of people aged 60 and over in rural districts of India and South Africa report control rates of 4%.^33^^,^^34^ A survey of adults in the former Soviet Union, who had been prescribed hypertension treatment, found that only 26% were taking it every day.^35^ A multicentre study of people aged 60 years and over in India and Bangladesh, conducted in 1999/2000, reported awareness rates of 45% and control of 10%.^36^ Levels of control in high-income countries are generally higher, but these also vary considerably, ranging from 20% in England to over 50% in the USA.^21^^,^^22^ The SAGE data show that awareness, treatment and control were usually higher for women and people at older ages. Few other studies specifically assess sex and age variations within older populations for awareness, treatment and control. The Chinese national survey does not find a strong age effect, with little overall variation in age deciles aged 45 years and over.^18^

Associations between wealth, education and health insurance status, on one hand, and prevalence, treatment and control, on the other, are more complex and challenging to interpret. Whereas the pooled data show that poorer groups are just as likely to experience hypertension as richer ones, there are varying patterns of social gradient across the six countries. Standard models of epidemiological transition posit that the strength and direction of social gradients will be related to a country’s general level of socio-economic development.^37^ The national level SAGE data do not fit neatly into this predicted pattern, and reveal complex variations across the five wealth quintiles. For example, in India only the wealthiest quintile is positively associated with prevalence, whereas in Mexico only the second poorest quintile is so associated. Similarly, in China higher education is negatively associated with prevalence, whereas in Ghana having no education is negatively associated. Results for awareness and the effective treatment of hypertension are more consistent, with urban groups performing better in five countries and the highest wealth quintile in four. The only clear exception to this trend is Mexico, which may reflect potential bias from the low response rates. Another explanation of high rates of rural awareness may be the effectiveness of health interventions targeting rural groups, notably Mexico’s Popular Health Insurance Programme, in screening and treating hypertension.^38^ For other countries and for the pooled data, health insurance coverage is often a poor predictor of treatment and control. In India, despite having the lowest health insurance coverage (4%), levels of awareness and treatment are both notably higher than in the other study countries. This may be due to disproportionately high prevalence among richer groups in India who are better placed to access effective treatment regardless of their insurance status. In the case of India’s poorest wealth quintile, only 5.3% of hypertensive people are controlled, compared with 23.5% of the richest. The lack of association between health insurance coverage and treatment or control at the national level among the SAGE countries may reflect large variations in the extent and quality of services offered by different schemes. Other studies have found these to be highly variable.^38^^,^^39^

Studies show that similar automated wrist devices to measure blood pressure compare well with gold-standard measurements using sphygmomanometers and trained health personnel.^13^^, ^^40^ A possible limitation of our study is that we relied on lay trained non-clinician interviewers for the measurement of blood pressure. Data suggest that trained personnel actually underestimate the prevalence of hypertension as compared with clinicians.^40^ Our classification of hypertension was based on an average of two measurements and not on more regular monitoring. This may have contributed to overestimation of hypertension prevalence. These two sources of opposing bias may result in our hypertension estimates being close to the true prevalence.

## Conclusions and policy implications

High prevalence among older adults in South Africa and Ghana raises the possibility that all countries across sub-Saharan Africa may already be experiencing globally unprecedented rates of hypertension. This corresponds with the results of a synthetic estimate of the global mean blood pressure trends which found highest levels in sub-Saharan Africa.^32^

National variations in hypertension do not correlate with economic and social development, based on indicators such as wealth, education and urbanization (Supplementary Table A3, available as Supplementary data at *IJE* online).This shows the need for a more nuanced appreciation of relationships between development and NCDs than is often made in the general literature.^2^^,^^41^ High BMI was a key determinant of national variations although, as with hypertension, BMI did not correlate with general development indicators at the national level. The reasons for these discrepancies have not been systematically researched and require further analysis.^42^

The SAGE findings indicate that hypertension affects poorer groups just as much as the rich, if not more. Even so, only 16% of hypertensive people in the wealthiest quintile had effectively controlled their condition. The failure to control hypertension cuts across all social strata, which may increase the political leverage to develop meaningful responses. Better access to healthcare among the urban population has a positive effect on controlling hypertension and may be a benefit of urbanization. As such, the simple causal link that is often made between urbanization and NCDs requires qualification.

The prominence of NCDs, and metabolic risk factors such as hypertension, in global health and development agendas has risen quickly. Nevertheless, there remains a large gap between discourse and policy practice. It has been estimated that NCDs accounted for only 3% of total global health assistance between 2001 and 2008.^43^ Given the close association between hypertension and BMI, interventions targeting diet and exercise should be given the highest possible priority. Salt restriction through voluntary food industry changes in food processing and advice to reduce salt intake should be promoted as a means of shifting the overall distribution of blood pressure downwards.^44^ However, the barriers against the rapid success of such interventions are formidable. These include resistance from powerful economic interests as well as cultural reluctance to embrace behaviour change.^3^^,^^45^ In the short term, the most effective strategy to reduce the burden of hypertension is through use of simple medication.^46^ Cost-effectiveness studies demonstrate the affordability of such interventions, although reaching at-risk groups with affordable treatment and persuading them to adhere to lifetime drug regimens still represents a significant challenge. This is demonstrated by the gap between awareness and control reported by the SAGE survey, and highlights the need for innovative delivery mechanisms. For example, in the case of rural populations in South Africa, there may be opportunities to link treatment of hypertension and other common NCDs with the monthly delivery of social pensions to villages.

More generally, interventions will require a reorientation of primary healthcare services towards the primary prevention and management of NCDs and the needs of older adults. As with other major epidemics such as HIV/AIDS, responding to the global crisis of hypertension requires multiple strategies including awareness raising, primary prevention and medication. If global and national efforts are not transformed with immediate effect, the potential consequences for the health and well-being of people in LMICs will be catastrophic.

## Supplementary Data

Supplementary data are available at *IJE* online.

## Funding

This work was supported by the National Institutes of Health (grants OGHA 04034785; YA1323-08-CN-0020; Y1-AG-1005-0 (R01-AG034479), which funded the WHO Study on global AGEing and adult health (SAGE) on which this analysis is based. Part of the analysis was funded by the Economic and Social Research Council (grant ES/K003526/1).

## Supplementary Material

Supplementary Data
