# Supplementary material for: Hypertension among older adults in low- and middle-income countries: prevalence, awareness and control
Source: Int J Epidemiol. 2014 Feb 6;43(1):116–28. doi: 10.1093/ije/dyt215 (PMC3937973; doi:10.1093/ije/dyt215)
Supplement: Supplementary Data [file supp_43_1_116__index.html]

Hypertension among older adults in low- and middle-income countries: prevalence, awareness and control — Hypertension among older adults in low- and middle-income countries: prevalence, awareness and control — Supplementary Data 

# Hypertension among older adults in low- and middle-income countries: prevalence, awareness and control

## Supplementary Data

files

**Files in this Data Supplement:**

- Supplementary Data - pdf file
