# Supplementary material for: Hypertension among older adults in low- and middle-income countries: prevalence, awareness and control
Source: Int J Epidemiol. 2014 Feb 6;43(1):116–28. doi: 10.1093/ije/dyt215 (PMC3937973; doi:10.1093/ije/dyt215)
Supplement: Supplementary Data [file supp_dyt215_ije-2013-03-0305.pdf]

Supplementary tables.

Table A1. SAGE response rates and missing data.

	China	Ghana	India	Mexico	Russian Fed.	South Africa
Target sample (n)	14,280	5,493	7,511	4,414	4,785	4,937
Complete/partial interview (n)	13,175	4,305	6,560	2,313	3,938	3,837
Response rate (%)	92.3	78.4	87.3	52.4	82.3	77.7
Blood pressure measurements recorded (n)	12,517	4,245	6,502	2,117	3,724	3,717
Blood pressure measurements response rate (%)	95	99	99	92	95	97
Missing education data for interviews with blood pressure measurement (n)	0	27	0	66	2	579
Missing BMI data for interviews with blood pressure measurement (n)	179	114	177	142	409	238
Missing smoking data for interviews with blood pressure measurement (n)	50	16	2	66	23	111
Missing alcohol data for interviews with blood pressure measurement (n)	61	22	1	66	19	118
Missing physical activity data for interviews with blood pressure measurement (n)	7	5	0	66	15	83
Missing income data for interviews with blood pressure measurement (n)	57	5	38	1	5	18
Missing insurance data for interviews with blood pressure measurement (n)	80	3	0	13	16	40

Table A2 Prevalence of effective treatment and adjusted model for effective treatment in hypertensive participants under treatment (Odds Ratios with 95% Confidence Intervals).

		Overall	China	Ghana	India	Mexico	Russian Federation	South Africa
		(n=6687)	(n=2470)	(n=395)	(n=497)	(n=438)	(n=1365)	(n=715)
Prevalence of effective treatment (%)								
Total		26.3 (25.3-27.3)	24.0 (22.3-25.7)	23.7 (19.6-27.8)	55.2 (50.9-59.5)	30.3 (26.2-34.4)	17.4 (15.5-19.3)	23.5 (20.8-26.2)
Male		28.3 (26.6-30.0)	27.6 (24.9-30.3)	26.2 (19.8-32.6)	55.4 (49.0-61.8)	21.1 (14.9-27.3)	17.5 (14.0-21.0)	20.5 (16.2-24.8)
Female		25.1 (23.8-26.4)	21.4 (19.3-23.5)	21.8 (16.5-27.1)	55.0 (49.3-60.7)	35.3 (30.0-40.6)	17.3 (15.0-19.6)	25.2 (21.7-28.7)
Odds Ratios								
Age (years)	[50-54)	1	1	1	1	1	1	1
	[55-59)	1.22 (1.00-1.50)	1.02 (0.71-1.47)	1.58 (0.62-4.05)	1.49 (0.81-2.73)	4.04 (1.26-12.9)	0.95 (0.55-1.63)	0.76 (0.40-1.42)
	[60-64)	1.11 (0.89-1.38)	0.86 (0.59-1.25)	0.68 (0.21-2.14)	1.50 (0.76-2.97)	1.28 (0.39-4.22)	1.81 (1.03-3.16)	0.37 (0.18-0.76)
	[65-69)	1.16 (0.94-1.44)	0.91 (0.63-1.32)	1.23 (0.46-3.26)	1.68 (0.84-3.34)	1.07 (0.32-3.54)	1.49 (0.85-2.64)	1.35 (0.75-2.43)
	[70+	0.93 (0.76-1.14)	0.81 (0.57-1.14)	1.04 (0.43-2.53)	1.78 (0.95-3.31)	1.28 (0.42-3.91)	0.82 (0.47-1.41)	0.65 (0.36-1.16)
Sex	Males	1	1	1	1	1	1	1
	Females	0.92 (0.81-1.04)	0.76 (0.61-0.94)	1.26 (0.72-2.21)	0.76 (0.47-1.23)	1.43 (0.83-2.44)	1.18 (0.83-1.68)	1.37 (0.89-2.12)
BMI	Normal	1	1	1	1	1	1	1
	Underweight	1.97 (1.46-2.65)	0.96 (0.49-1.87)	0.78 (0.25-2.40)	1.97 (1.10-3.54)	13.2 (0.23-743.5)	<0.001 (<0.001- >999)	3.54 (1.30-9.65)
	Overweight	0.76 (0.66-0.87)	0.70 (0.57-0.86)	0.38 (0.20-0.72)	1.33 (0.82-2.15)	1.18 (0.61-2.26)	0.59 (0.39-0.90)	0.94 (0.49-1.80)
	Obese	0.45 (0.38-0.53)	0.58 (0.40-0.85)	0.29 (0.13-0.64)	1.90 (0.80-4.52)	0.82 (0.41-1.66)	0.28 (0.18-0.44)	1.05 (0.58-1.91)
Health insurance	Insured	1	1	1	1	1	1	1
	Uninsured	2.34 (2.04-2.69)	1.30 (0.97-1.74)	0.91 (0.54-1.53)	0.68 (0.35-1.30)	0.51 (0.27-0.96)	4.43 (0.33-59.6)	0.99 (0.62-1.56)
Location	Urban	1	1	1	1	1	1	1
	Rural	0.59 (0.51-0.67)	0.30 (0.22-0.39)	0.58 (0.30-1.13)	0.72 (0.47-1.12)	1.36 (0.79-2.33)	0.48 (0.32-0.73)	0.82 (0.52-1.32)
Education	Primary	1	1	1	1	1	1	1
	None	1.34 (1.12-1.61)	1.03 (0.76-1.40)	0.67 (0.32-1.41)	1.60 (0.96-2.67)	2.08 (1.11-3.89)	0.23 (0.01-7.53)	1.82 (1.09-3.03)

	Secondary/Higher	0.98 (0.85-1.14)	1.23 (0.98-1.55)	0.56 (0.26-1.16)	0.92 (0.53-1.59)	0.94 (0.49-1.83)	0.68 (0.38-1.20)	1.77 (1.11-2.82)
Wealth quintile	Poorest	1	1	1	1	1	1	1
	Q2	1.08 (0.85-1.36)	1.04 (0.70-1.53)	0.47 (0.10-2.10)	2.07 (0.75-5.71)	0.85 (0.38-1.91)	0.85 (0.47-1.54)	3.37 (1.60-7.08)
	Q3	1.13 (0.90-1.42)	1.04 (0.71-1.52)	0.58 (0.16-2.05)	1.04 (0.40-2.71)	1.69 (0.76-3.73)	0.77 (0.43-1.39)	1.92 (0.90-4.08)
	Q4	1.51 (1.21-1.87)	1.06 (0.73-1.54)	0.65 (0.19-2.16)	1.48 (0.60-3.63)	1.73 (0.79-3.76)	1.41 (1.78-2.53)	4.01 (1.90-8.43)
	Richest	1.55 (1.25-1.92)	0.84 (0.58-1.24)	1.41 (0.43-4.58)	1.32 (0.53-3.25)	1.22 (0.57-2.60)	1.37 (0.76-2.48)	3.04 (1.45-6.35)
Alcohol consumption	Life time abstainers	1	1	1	1	1	1	1
	Non heavy drinker	1.00 (0.84-1.20)	0.85 (0.62-1.18)	2.98 (1.55-5.70)	0.58 (0.22-1.55)	0.72 (0.26-2.00)	1.55 (1.08-2.24)	0.28 (0.10-0.81)
	Infrequent heavy drinkers	1.20 (0.76-1.89)	0.66 (0.22-1.96)	-	-	0.14 (0.03-0.60)	3.87 (1.55-9.68)	1.34 (0.28-6.33)
	Frequent heavy drinkers	0.69 (0.36-1.32)	1.23 (0.61-2.47)	<0.001 (<0.001 - >999)	<0.001 (<0.001 - >999)	-	0.07 (<0.001-53.4)	<0.001 (<0.001 - >999)
Physical activity	High	1	1	1	1	1	1	1
	Moderate	0.99 (0.85-1.14)	1.10 (0.87-1.39)	1.16 (0.52-2.56)	0.76 (0.46-1.25)	1.28 (0.69-2.39)	0.81 (0.53-1.23)	2.04 (1.01-4.12)
	Low	0.72 (0.62-0.83)	0.91 (0.71-1.17)	2.72 (1.40-5.26)	0.40 (0.25-0.65)	0.94 (0.53-1.66)	0.63 (0.41-0.95)	1.50 (0.89-2.55)

Table A3. Age-adjusted prevalence of hypertension in SAGE participants over the age of 50 years [95%CI] and selected national development indicators.

	SAGE participants			National development indicators				
	Total	Male	Female	Population aged 50+ (% of total) 2010	Per capita GNI (2008) (US$)*	% urban (2005)*	Adult literacy rate 15+ (2007) (%)*	Human Development Index (2010)[§]
**Percent Hypertensive (95% CI)**								
**China**	59.5 (58.4-60.6) (n=12295)	58.8 (57.6-60.0) (n=6047)	60.1 (58.9-61.3) (n=6248)	24.4	6,020	42.5	93	0.663
**Ghana**	57.1 (55.1-59.1) (n=4220)	54.6 (52.5-56.7) (n=2211)	59.9 (57.8-62.0) (n=2009)	11.5	1,430	47.8	65	0.464
**India**	32.3 (30.3-34.3) (n=6250)	30.3 (28.7-31.9) (n=3226)	35.0 (33.3-36.7) (n=3024)	15.9	2,960	28.7	66	0.519
**Mexico**	58.2 (55.4-61.0) (n=2095)	55.2 (52.1-58.3) (n=976)	60.9 (58.0-63.8) (n=1119)	17.4	14,270	76.3	93	0.750
**South Africa**	77.9 (76.4-79.4) (n=3634)	74.7 (72.6-76.8) (n=1596)	80.3 (78.6-82.0) (n=2038)	15.4	9,780	59.3	88	0.597
**Russian Federation**	71.7 (69.9-73.5) (n=3491)	65.9 (63.4-68.4) (n=1372)	74.5 (72.6-76.4) (n=2119)	32.8	15,630	72.9	100	0.719

*Sources: World Urbanization Prospects: The 2009 Revision Population Database http://esa.un.org/unpd/wpp/index.htm;
World Development Report 2010 http://econ.worldbank.org/wdr/

[§] This index combines life expectancy, years of schooling and per capita income (Human Development Report 2010).
